# HPV-Related Cervical Cancer and Extracellular Vesicles

**DOI:** 10.3390/diagnostics12112584

**Published:** 2022-10-25

**Authors:** Magdalena Kaczmarek, Monika Baj-Krzyworzeka, Łukasz Bogucki, Magdalena Dutsch-Wicherek

**Affiliations:** 1 Department of Endoscopic Otorhinolaryngology, Centre of Postgraduate Medical Education (CMKP), 01-813 Warsaw, Poland; 2 Department of Clinical Immunology, Institute of Paediatrics, Jagiellonian University Medical College, 30-663 Kraków, Poland

**Keywords:** extracellular vesicles (EVs), HPV infection, cervical cancer

## Abstract

Cervical cancer is the fourth most common type of cancer in females worldwide. Infection with a human papillomavirus is crucial to the etiopathogenesis of cervical cancer. The natural trajectory of HPV infection comprises HPV acquisition, HPV persistence versus clearance, and progression to precancer and invasive cancer. The majority of HPV infections are cleared and controlled by the immune system within 2 years, but some infections may become quiescent or undetectable. The persistence of high-risk HPV infection for a longer period of time enhances the risk of malignant transformation of infected cells; however, the mechanisms responsible for the persistence of infection are not yet well-understood. It is estimated that 10–15% of infections do persist, and the local microenvironment is now recognized as an important cofactor promoting infection maintenance. Extracellular vesicles (EVs) are small membrane vesicles derived from both normal cells and cancer cells. EVs contain various proteins, such as cytoskeletal proteins, adhesion molecules, heat shock proteins, major histocompatibility complex, and membrane fusion proteins. EVs derived from HPV-infected cells also contain viral proteins and nucleic acids. These biologically active molecules are transferred via EVs to target cells, constituting a kind of cell-to-cell communication. The viral components incorporated into EVs are transmitted independently of the production of infectious virions. This mode of transfer makes EVs a perfect vector for viruses and their components. EVs participate in both physiological and pathological conditions; they have also been identified as one of the mediators involved in cancer metastasis. This review discusses the potential role of EVs in remodeling the cervical cancer microenvironment which may be crucial to tumor development and the acquisition of metastatic potential. EVs are promising as potential biomarkers in cervical cancer.

## 1. Cervical Cancer and HPV Infection

Cervical cancer is the fourth most common type of cancer in females worldwide [1]. Infection with a human papillomavirus (HPV) plays a pivotal role in the etiopathogenesis of cervical cancer. Human papillomaviruses belong to the *Papillomaviridae* family, a small group of nonenveloped viruses (of which over 200 types have been identified), with a genome in the form of a circular double-stranded DNA. HPV infection is the most common type of sexually transmitted disease. HPV causes about 5% of all cases of cancer globally, and approximately 3–3.5% of these cases are caused by HPV16 [2,3,4]. High-risk HPV (hr-HPV) mucosal viruses (predominantly types 16, 18, 31, 33, and 35) have been related with various cancers, including cervical cancer [5]. HPVs are characterized by tropism to the skin and the mucosa; the function of high-risk HPVs is primarily realized through the E6 and E7 oncoproteins [5].

Prominent expression of the E6 and E7 oncoproteins deregulates the cell cycle, enhances cell division, inhibits apoptosis, and promotes the gathering of genetic errors due to ineffective DNA repair, leading to the development of malignancies. The E6 oncoprotein creates a complex with the E6AP ubiquitin ligase and the p53 protein, blocking apoptosis by proteasomal degradation. The E7 oncoprotein connects to pRB (which functions as a tumor suppressor), releases the E2F transcription factor from the pRB/E2F complex, and leads to cell cycle deregulation. Oncoproteins E6 and E7 also affect cytokine expression and activation of signaling pathways (PI3K/AKT, Wnt, and Notch) [6,7,8]. The natural trajectory of HPV infection comprises HPV infection persistence or healing, HPV genome integration, and progression to precancer and invasive cancer [9]. The majority of HPV infections (about 90%) are cleared and controlled by the immune system within 2 years; however, some infections may become quiescent (latent) or undetectable [10]. The persistence of hr-HPV infection for longer than 6 months enhances the risk of malignant transformation of the infected cells [1], but the mechanisms responsible for infection persistence are not yet well-understood. It is estimated that 10–15% of infections persist [1], and the local microenvironment is now recognized as an important cofactor promoting infection maintenance [11,12,13].

## 2. Extracellular Vesicles (EVs): Nomenclature and Classification

Extracellular vesicles (EVs) are spherical membrane-derived particles that are released by almost all types of cells, including tumor cells. EVs are produced throughout the normal lifespan and during certain conditions that promote activation such as viral infection, stress, and proliferation. According to the nomenclature established by the ISEV (International Society of Extracellular Vesicles) in 2018 [14], EVs are classified according to the subtypes that mirror their physical characteristics, biochemical composition, or origin. EVs are also grouped according to their size into small EVs (<100 or 200 nm, previously called exosomes) or medium/large EVs (>200 nm, previously known as mi-crovesicles or ectosomes). The density of EVs is roughly 1.11–1.2 g/mL [15]. The classification is based on the biochemical composition of EVs and includes the expression of proteins derived from the endolysosomal compartment, such as tetraspanins (CD9, CD53, CD63, CD81, and CD82); proteins of the ESCRT complex (Alix, TSG101); chaperones; and a variety of plasma membrane-derived molecules of parental origin. Exosomes are a type of EVs formed as intraluminal vesicles that are released to the extracellular space after the fusion of late endosomes with the plasma membrane [16]. Microvesicles/ectosomes are released by the budding of the plasma membrane and may share its characteristics. EVs released from dying cells are called apoptotic bodies; however, their relative size (from 50 nm to 2 µm) and composition differ from those of other EVs [17]. Recently, very small particles (<50 nm) have been described and named exomeres [18].

## 3. Cargo and Fate of EVs

EVs carry lipids, proteins, carbohydrates, RNA, and DNA derived from the donor cells [19,20,21,22,23,24]. Zhang reported more than 50,000 proteins, 164,000 mRNAs, and 12,000 miRNAs present in EVs of different origins [24]. The data concerning the ssDNA and dsDNA content of EVs are limited [25,26].

The cellular components of EVs do not simply reflect the parental cell content. Instead, it appears that EVs are actively loaded with cargo [26] according to the cell activation status and EV subtype. For example, EVs derived from cervical cancer cell lines (mostly HPV+) are enriched with miRNAs in comparison to their parental cells [27]. However, there is no proof of HPV miR expression within these EVs. Protection of the cargo in the lumen of membrane vesicles, which are stable in biological fluids, is a conserved phenomenon for the transfer between cells. In this context, EVs seem to have many traits in common with viruses; for example, both originate from the endosomal system (viruses enter it via endocytosis or membrane fusion) and are surrounded by a lipoprotein membrane. EVs, particularly small EVs, released from virus-infected cells may contain viral proteins and nucleic acids, which are loaded into them in the endosome via the ESCRT pathway or interaction with tetraspanins [28].

The presence of virions in small EVs has been noted through an elegant theory called “The Trojan Horse”, which uses HIV as an example [29]. The Trojan Horse hypothesis assumes that viruses can use the exosome biogenesis pathway to release virus-loaded vesicles (infectious) in exosomal vesicles. Interestingly, this phenomenon is used by both enveloped (e.g., HIV, influenza, and SARS-CoV-2) and nonenveloped (HAV and HPV) viruses. In the latter case, the replicative viral structures can be “quasi-enveloped” (e.g., “cloaked” inside EVs) [30]. EVs loaded with viruses may be directly infective as proven for HBV, HIV, and EBV [31] in parallel with replicative viral particles. The examples of virus-derived components detected in EVs are presented in Table 1.

On the other hand, viruses and small EVs are similar in size (<400 nm) and may be copurified from the infected cells (using the physical properties of the vesicles only), which makes research of the different types of vesicles difficult. Virus particles are similar in density (the density of HIV-1 particles is 1.16–1.18 g/mL) to EVs [32]. Ludwig et al. revealed that small EVs isolated from HPV+ and HPV− cell lines are morphologically indistinguishable [33]; however, the differences in their miR profiles are significant [33]. Tong et al. presented a total of 32 miRNAs that were differentially expressed between HPV+ and HPV− exosomes; miR-1306-5p, -193b-5p, -92b-3p, -92b-5p, -365b-3p, -125a-3p, and let-7b-5p were enriched with HPV+ exosomes in comparison to HPV– exosomes [27].

All types of EVs transport their content from the parental to the recipient cells, constituting a mode of “cell-to-cell” communication [20,34,35,36,37]. EVs bind cells and are either engulfed by them or are fused with the membrane of recipient cells. Endocytosis has been proposed as the primary mechanism of EV uptake from the microenvironment; however, the specific type of this phenomenon is debatable (endocytosis mediated by clathrins, caveolin-dependent; lipid raft-mediated endocytosis; phagocytosis or micropinocytosis) and depends on the recipient cells [38,39]. Cells can internalize EVs at the same time through different mechanisms with different efficiency.

## 4. Extracellular Vesicles, HPV Infection, and Cervical Cancer

EVs play an important role in both physiological and pathological processes. They have also been identified as one of the mediators involved in cancer metastasis [61,62,63]. The remodeling of the cancer microenvironment is thought to be crucial to the development of the tumor and the acquisition of metastatic potential. Crosstalk between cancer processes and the cancer microenvironment enables microenvironment remodeling. It has been discovered that tumor-derived EVs can be captured by stromal cells, leading to the conversion to a tumor-associated microenvironment prone to tumor development [64]. In a study of highly metastatic breast cancer cells, exosomes were found to participate in stromal cell education [65]. In a study using fluorescent protein imaging, Liang et al. provided direct evidence of the transfer of exosomes from highly metastatic breast cancer cells to adjacent cancer cells and lung tissue cells both in vitro and in vivo. These findings confirmed that EVs derived from metastatic cancer cells are involved in the education of stromal cells through an intercellular communication pathway whereby fibroblast exosomes mobilize autocrine Wnt–PCP signaling to drive the invasive behavior of cancer cells [66].

Zhang et al. demonstrated that a coculture of Langerhans cells with EVs derived from oncoprotein E7-expressing cells suppresses surface CD40 expression and the intracellular proinflammatory cytokine IL-12p40 subunit in Langerhans cells; this coculture also inhibits cytotoxic T cell response. Such an observation proves that EVs derived from HPV-infected cells can suppress the local immune response, thus participating in the persistence of the HPV infection [13].

Khan et al. reported that an HPV18-infected cell line (HeLa) derived from cervical adenocarcinoma tissues released EVs containing survivin, a member of the inhibitory apoptosis protein family; the mRNA encoding survivin has been detected in HeLa cell lines [67,68]. Khan et al. found that extracellular survivin can enhance cellular proliferation and survival as well as tumor cell invasion when released from exosomes to the extracellular space in patients with cervical cancer (HeLa) [67]. Moreover, the amount of survivin increased significantly in the basal state, after proton irradiation, and in the stress-induced state. Survivin may also be involved in the induction of chemoresistance in other receptor cells [68]. It was concluded that survivin liberated to the microenvironment by EVs may play an important protumorigenic role [63,68].

Honegger et al. assessed the E6/E7 presence in extracellular vesicles originating from the HeLa cell line, but these proteins were not found [69]. Ranjit et al. examined the E6/E7 proteins in extracellular vesicles derived from the CaSki cell line. In this study, E6 was identified, while the E7 oncoprotein was not [51]. The delivery of the E6/E7 oncogenes by EVs affects the miRNA profile of cervical cancer cell lines. For example, E6/E7 are required to express miR-17-92 which downregulates the expression of the antiproliferative p21 gene [70]. EVs are absorbed by cancer cells, which thereby gain more proliferative properties, thus becoming resistant to therapy. This effect may arise through the transference of oncogenes and the induction of microenvironment remodeling. The composition of EVs may be modified by virus-infected cancer cells, with the enrichment of oncogenes and factors such as survivin.

Oncogenic HPV DNA was demonstrated to be transported from cancer cells to fibroblasts [71]. Honegger et al. showed that inhibition of the endogenous HPV18 E6/E7 oncogene expression leads to alterations in the content and number of EVs released from HeLa cervical cancer cells. Intracellular survivin protein levels are strongly lowered on the suppression of the endogenous HPV18 E6/E7 expression, suggesting that viral oncogenes play an important role in the maintenance of survivin protein accumulation in HPV-positive cancer cells [69]. Moreover, the authors observed that the increase in small EV release in HPV-positive cervical cancer cells is attended by the initiation of senescence due to the suppression of the endogenous viral oncogene expression as a senescence trigger [69]. They also noted that cervical cancer cells release a similar number of small EVs [72,73]. Furthermore, Saha and Liang showed that Wnt proteins occur in vivo together with some exosomal markers; thus, WNT proteins might be transferred by small EVs [66,74]. The authors also showed that circulating small EVs derived from patients with cervical cancer are able to functionally affect fibroblast differentiation by Wnt2B via small EV-dependent secretion. As a result, the profile of stromal fibroblasts was changed into that of cancer-associated fibroblasts through the WNT/beta catenin signaling pathway, thus increasing the migration and proliferation of these cells. This process influences the remodeling of the cancer microenvironment [66].

Zhang et al. found that HPV16 E7 increased EV production from murine and human keratinocytes. Moreover, the EVs shed from these cells exerted an immune regulatory function on the neighboring Langerhans cells. The Langerhans cells and T cells cocultured with these EVs suppressed antigen-specific cytotoxicity [13].

Iuliano et al. analyzed the expression of inflammatory mediators in HPV-positive cells in primary human foreskin keratinocytes and keratinocytes transduced by E6 and E7 from mucosal HPV16 or cutaneous HPV38 genotypes. They demonstrated that the expression of inflammatory cytokines, such as IL-1α, IL-1β, and IL-6, was downregulated in K16 and K38 cells, while TNF-α was upregulated in the same cells. Apart from the E6 and E7 oncoproteins’ influence on p53 and pRB, they were shown to repress NF-κB signaling. It has been suggested that NF-κB suppression mediated by viral oncogenes plays a key role in the induction of escape from immune recognition. In addition, it has been shown that HPV can alter the production of chemokines and influences leukocyte trafficking to the skin by suppressing CCL20/MIP-3α. The authors further observed that HPV-positive cells released EVs derived from CCL20/MIP-3α mRNA [75]. HPV16 E6 and E7 suppressed the expression of CCL2/MCP-1 in cervical epithelial cells as well as in epidermal cells [76]. HPV E6 and E7 can alter not only microRNA expression, but the mRNA content within EVs [75].

Thakur et al. demonstrated that exosomes derived from tumors with double-stranded DNA and exosomal DNA represent the entire genome and reflect the mutational status of the parental tumor cells. The authors underlined the potential role of these EVs as a circulating biomarker for the early detection of cancer and metastases [77]. Moreover, Thippabhotla et al. observed extracellular vesicles derived from 3D and 2D cultures. The molecular load of the EVs and the form of secretion varied, reflecting their origin cell status independently of the culture or conditions [78]. This observation confirms that the development of EVs is an active process reflecting the changes in the microenvironment or in the cells of origin; miRNA carried by EVs is transferred to the recipient cells (neighboring or distant cells) and is responsible for the alteration of their function.

## 5. The Potential Role of Extracellular Vesicles as Biomarkers in Patients with Cervical Cancer

Cervical cancer-derived EVs seem to be a hallmark of the important steps in cancer progression, namely angiogenesis, migration, and invasion. Identification of the molecules contained in EVs would allow their use as markers of cancer disease progression, viral integration, and infection. Apart from identifying the proteins mentioned above, the promising results direct attention to microRNAs (miRNAs), the noncoding RNA molecules that regulate gene expression. Numerous oncogenic microRNAs have been observed in association with cervical cancer cells [79,80]; miR-21 and miR-146a have been found to promote cell growth, migration, and invasion in patients with cervical cancer [81,82]. Liu et al. studied EVs purified from cervicovaginal lavage samples obtained from cervical cancer patients. They demonstrated that these EVs contained high levels of microRNA-21 and microRNA-146a in comparison to the samples obtained from noncancer patients [83]. HPV infection seems to contribute to the upregulation of both miR-21 and miR-146 in cervical tissue. The highest expression levels of miR-21 and miR-146a were observed in the EVs found in the cervicovaginal lavage of both the cancer patients and the HPV-positive healthy women. Cervical cancer-associated miR-21 and miR-146a were secreted from the cancer tissue within cervicovaginal exosomes, indicating their potential to become a cervical cancer biomarker [83].

Higher levels of both let-7d-3p and miR-30d-5p were identified in EVs derived from the plasma of cervical cancer patients compared to the levels found in healthy volunteers and precancerous patients. The authors concluded that exosomal let-7d-3p and miR-30d-5p are valuable diagnostic biomarkers for the noninvasive screening of cervical cancer and its precursors [84].

Pan et al. noted the downregulation of both MeCP2 and MBD2 mRNA expression in cervical cancer tissues. MBD2 and MeCP2 are targets of miR-221 and miR-222, both of which are upregulated in cervical cancer tissue and cell lines. They observed that upregulated miR-221/222 promote cervical cancer by repressing MBD2 and MeCP2 [85]. In addition, the expression of miR-221-3p was detected in exosomes derived from cervical cancer cell lines [86]; microRNA-221-3p was shown to correlate with microvascular density in cervical squamous cell carcinoma patients. It was observed to be significantly overexpressed in human cervical squamous cell carcinoma tissues derived from patients with lymph node metastases compared to patients without metastases. The phenomenon of epithelial–mesenchymal transition (EMT) was promoted by miR-221-3p expression; it stimulated cell migration and invasion in vitro and the promotion of lymph node metastases in vivo. MiRNAs are regulated by transcription factors; twist homolog 2 (TWIST2) was the main transcription factor connecting to miR-221-3p. The inhibition of miR-221-3p was found to reduce the promotion of EMT and decrease cell migration and invasion mediated by TWIST2 [87]. Wei et al. discovered EVs in cervical cancer patients that transport miR-221-3p from cancer cells to vessel endothelial cells and promote angiogenesis by downregulating thrombospondin-2 (THBS2). The authors concluded that cervical cancer-derived exosomal miR-221-3p could potentially become a diagnostic biological marker and a therapeutic target for cervical cancer progression [88].

MiR-125a-5p has been identified in normal tissue. In breast cancer tissue, hepatocellular cancer tissue, and their cell lines [89], miR-125a-5p had a decreased expression [90]. In lung cancer patients, an upregulation of miR-125a-5p induced cancer cell apoptosis through the activation of p53 [91]. The inhibition of miR-125a-5p in hepatocellular cancer patients increased the expression of MMP11 and the vascular endothelial growth factor A (VEGFA) protein [92]. Exosomal miR-125a-5p expression levels were examined in the tissues of cervical cancer patients and found to be significantly lower than those in the healthy controls. Thus, exosomal miR-125a-5p seems to be a potential biomarker for cervical cancer diagnosis [93].

Long noncoding RNAs were identified in cervicovaginal lavage-derived EVs from cervical cancer patients and in HeLa-derived exosomes (CC-NDA1, HOTAIR, TUG1, MALAT1, MEG3, GAS5.132, EXOC7, lincRNA-p21, and HNF1A-AS1). These exosomes are involved in cancer proliferation, metastasis, invasion, apoptosis, migration, and EMT and may impact resistance to treatment; thus, they could become noninvasive biomarkers for the early diagnosis of cervical cancer [94].

A novel therapy could target the extracellular survivin released by the exosomes described above. As Li et al. summarized, there are five potential therapeutic strategies that involve survivin: inhibitors disrupting survivin interactions with its partner proteins; inhibitors affecting survivin homodimerization; inhibitors decreasing survivin gene transcription; inhibitors inducing survivin mRNA degradation; and survivin-based cancer immunotherapy [95].

Virus-modified EVs may participate in the remodeling of the cancer microenvironment into proangiogenic and immunosuppressive, promoting cell migration and invasion by transferring oncogenic factors to the adjacent cells [63,96]. Potentially, EVs obtained by a noninvasive method, such as biopsy, might serve as a biomarker of cancer advancement [63,96].

## Figures and Tables

**Table 1:** Virus-derived components in extracellular vesicles.

Virus	Viral Components Detected in EVs	Source of Samples	Reference
HTLV-1	Tax	HTLV-1^+^ cell lines C8166-45 and MT2	Jaworski, 2014 [40]
HIV-1	Unspliced RNA TAR RNA	HIV-1-infected cell lines Patients’ sera	Columba Cabezas, 2013; Narayanan, 2013 [41,42]
	Nef, Gag	HIV-infected Jurkat cell line Patients’ plasma	Madison, 2015; Fang, 2007; Raymond, 2011 [43,44,45]
	vmiR-88, vmiR-99	Patients’ plasma	Bernard, 2014 [46]
CMV	gB protein	CMV-infected HUVEC cells	Walker, 2009 [47]
HSV1	miR-H5, miR-H3, miR-H6	HSV-infected cell lines	Kalamvoki M et al. [48]
EBV	LMP1 (latent membrane protein 1)	EBV-infected cell lines	Flanagan, 2003 [49]
	BGLF2 protein	EBV-infected cell lines	Sato, 2022 [50]
HPV	E6/E7 oncogenes HPV DNA	Cervical cancer cell lines, cervical scrape samples	Ranjit, 2020; Kannan, 2017; Mata-Rocha, 2019; Ludwig, 2018 [33,51,52,53]
HCV	RNA (complete genome) miR-122 HCV core proteins	Patients’ sera HCV-infected cell lines	Longatti, 2015; Bukong, 2014; Ramakrishnaiah, 2013 [54,55,56]
HBV	RNA HBV miRNA HBV proteins	HepG2 cell line Patients’ sera Patients’ sera	Kouwaki, 2016; Yang, 2017a; Yang, 2017b [57,58,59]
HAV	RNA Viral protein pX	HAV-infected cell lines	Longatti, 2015; Jiang, 2020 [54,60]

## Data Availability

Not applicable.
